# Affinities to Oxaliplatin: Vitamins from B Group vs. Nucleobases

**DOI:** 10.3390/ijms231810567

**Published:** 2022-09-12

**Authors:** Beata Szefler, Przemysław Czeleń, Kamil Wojtkowiak, Aneta Jezierska

**Affiliations:** 1 Department of Physical Chemistry, Faculty of Pharmacy, Collegium Medicum, Nicolaus Copernicus University, Kurpińskiego 5, 85-096 Bydgoszcz, Poland; 2 Faculty of Chemistry, University of Wrocław, F. Joliot-Curie 14, 50-383 Wrocław, Poland

**Keywords:** Oxaliplatin, platinum-based drugs, colorectal cancer, gastric cancer, esophageal cancer, cancer treatment, vitamins from B group, UV-Vis, DFT, TD-DFT, PCM

## Abstract

Oxaliplatin, similar to Cisplatin, exhibits anticancer activity by interacting with DNA and inducing programmed cell death. It is biotransformed through a number of spontaneous and non-enzymatic processes. In this way, several transient reactive species are formed, including dichloro-, monochloro-, and diaqua-DACH platin, which can complex with DNA and other macromolecules. The molecular level suggests that such interactions can also take place with vitamins containing aromatic rings with lone pair orbitals. Theoretical and experimental studies were performed to investigate interactions of vitamins from the B group with Oxaliplatin, and the results were compared with values characterizing native purines. Quantum-chemical simulations were carried out at the B3LYP/6-31G(d,p) level, with the LANL2DZ basis set representing atomic orbitals of platinum atom, and at the MN15/def2-TZVP levels of theory with the use of Polarizable Continuum Model (IEF-PCM formulation) and water as a solvent. Additionally, time-dependent density functional theory (TD-DFT) was employed to study molecular properties in the electronic excited state. Interactions of vitamins and Oxaliplatin were investigated using UV-Vis spectroscopy. Values of the free energy (ΔG_r_) indicate spontaneous reactions with monoaqua [PtH_2_OClDACH]^+^ and diaqua [Pt(H_2_O)_2_DACH]^2+^ derivatives of Oxaliplatin. However, diaqua derivatives were found to be preferable. The free energy (ΔG_r_) values obtained for vitamins from the B group indicate lower affinity of Oxaliplatin compared with values characterizing complexes formed by guanine, adenine, and cytosine. The exception is the monoaqua form of vitamin B1 (thiamine) at the MN15/def2-TZVP levels of calculations. An application of atoms in molecules (AIM) theory revealed non-covalent interactions present in the complexes studied. The comparison of computed and experimental spectroscopic properties showed a good agreement.

## 1. Introduction

**Figure 1:**
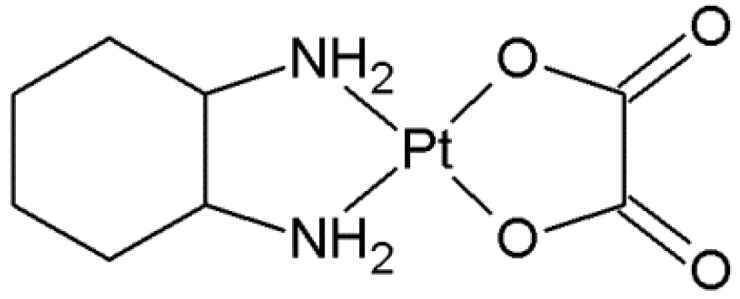
Structure of Oxaliplatin.

Oxaliplatin (Figure 1) is a complex compound of platinum in the second oxidation state with 1,2-diaminocyclohexane and an oxalate group (DACH). It is a cytostatic and alkylating drug used in chemotherapy of malignant tumors, mainly in the treatment of colorectal cancer, in combination with fluoropyrimidines [1,2,3,4,5,6,7,8,9,10,11,12,13]. A randomized study suggests the equivalence of Cisplatin and Oxaliplatin in the treatment of gastric or esophageal cancer [14,15]. In the preclinical studies, Oxaliplatin has demonstrated efficacy against a broad spectrum of investigated tumors, including some Cisplatin- and Carboplatin-resistant cell lines [16,17,18,19,20,21,22,23].

**Figure 2:**
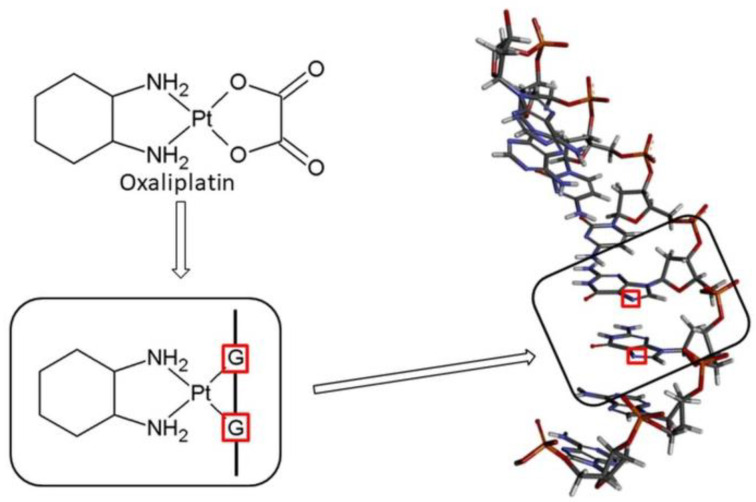
Pathway of Oxaliplatin and anticancer activity.

**Figure 3:**
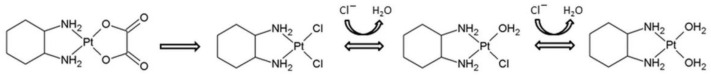
Biotransformation pathway of Oxaliplatin.

The main mechanism of action of the drug is based on the DNA damage (Figure 2), [24,25]. Similar to Cisplatin, Oxaliplatin binds to DNA, leading to GG intra-strand crosslinks [1]. Cancer cell apoptosis is caused by the DNA damage and blockage of its synthesis. After entering the cell, the drug approaches the cell nucleus, where it is directed to the sites rich in guanine, adenine and cytosine. Next, it interacts with the nitrogen atom (N_7_) forming a monoadduct, and then a diadduct (Figure 2 and Figure 3) [26]. This results in the formation of intra-chain and inter-chain cross-link bonds, and bonds between the DNA and the proteins (Figure 2). The DNA replication and transcription are stopped, followed by the cell apoptosis [27]. 

Oxaliplatin is biotransformed through a number of spontaneous and non-enzymatic processes [28,29,30,31]. Nucleophiles, such as Cl^−^, HCO_3_^−^, and H_2_O molecules, participate in these reactions (Figure 3) [30,32,33]. In this way, several transient reactive species are formed, including dichloro-, monochloro-, and diaqua-DACH platin, which can complex with DNA and other macromolecules [30,32,33].

**Figure 4:**
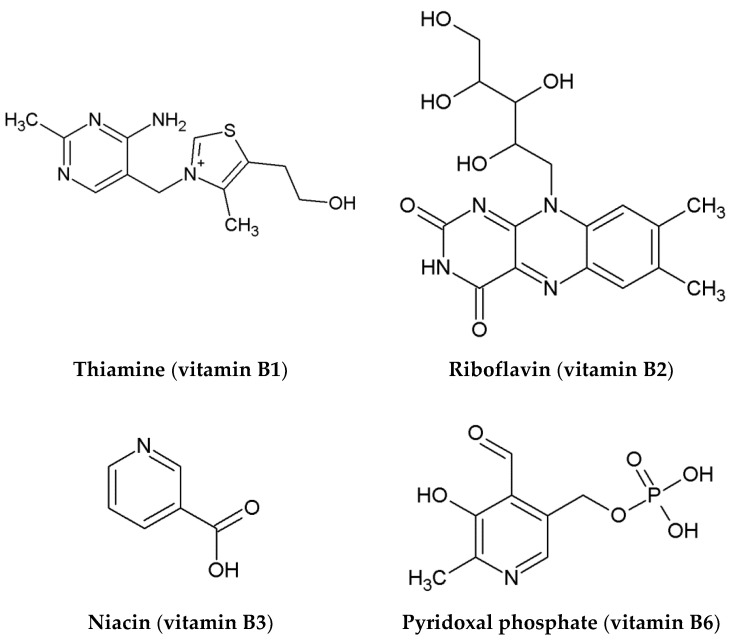
B vitamins chosen for the current study. The N_1_ and N_7_ atoms are indicated.

Oxaliplatin belongs to the same group of Pt (II) derivatives used in cancer treatment, and it has a similar mechanism of action when compared to Cisplatin. For the activation of relatively inert platinum (II) complexes, the hydrolysis of Oxaliplatin to monoaqua form and diaqua complexes is necessary for the replacement of chloride ligands with water molecules (Figure 3) [34]. In this way, Oxaliplatin can interact with DNA and induce programmed cell death (Figure 2). Therefore, it can be assumed (analogously to Cisplatin) that the presence of other compounds possessing aromatic rings with electron lone pairs, such as N_7_ or/and N_1_ atoms, influences the therapeutic effect of the chemotherapeutic [24,25]. During chemotherapy, patients often consume juices rich in niacin (vitamin B3), pyridoxal phosphate (vitamin B6), riboflavin (vitamin B2), and thiamine (vitamin B1) [24,25]. The structures of selected vitamins B are presented in Figure 4. Therefore, on the basis of our previous findings, which clearly showed the possibility of forming complexes of B vitamins with nucleobases [24,25], it can be assumed that the presence of B vitamins could reduce the therapeutic effect of the Oxaliplatin [24]. 

The theoretical and experimental studies of the interactions of the N_7_ nitrogen atom of B vitamins (and, in addition, the N_1_ nitrogen atom in the case of thiamine) with Oxaliplatin have been performed. The comparison of interactions of the studied drug with the nucleobases, such as adenine, guanine, and cytosine, which occur during chemotherapy, have been carried out. Finally, our results were compared with earlier obtained data for Cisplatin and Carboplatin [24,25].

## 2. Results and Discussion

### 2.1. Energetic and Electronic Structure Characterization in the Electronic Ground and Excited States (DFT and TD-DFT Study)

**Figure 5:**
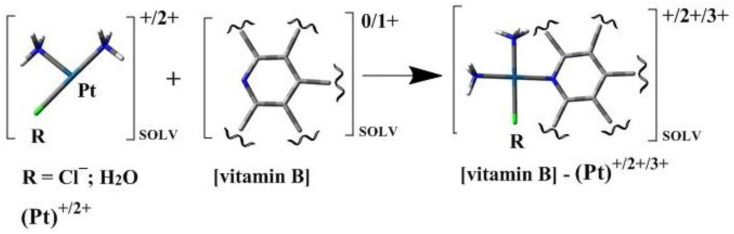
Scheme of complexes of B vitamins with Pt-mono and Pt-diaqua complexes.

Monoaqua [PtH_2_OClDACH]^+^ and diaqua [Pt(H_2_O)_2_DACH]^2+^ derivatives of Oxaliplatin, with vitamins from the B group such as thiamine (vitamin B1), riboflavin (vitamin B2), niacin (vitamin B3), and pyridoxal phosphate (vitamin B6) were studied (see Figure 4 and Figure 5). All these vitamins are present in carrot and beet juices, which are often consumed by patients during chemotherapy.

Because vitamins from the B group have a purine and/or pyrimidine rings with lone pair orbitals analogous to N_7_ in purine, they have a structure similar to native DNA purines and can freely react with Oxaliplatin. The vitamin B1 (thiamine), in contrast to other vitamins, has the second nitrogen atom in the position N_1_, which could also act as a possible reaction center (Figure 4).

As is shown in Table 1, Table 2, Table 3 and Table 4, the estimated values of Gibbs free energy of reaction ΔG_r_ are negative, not only for guanine, adenine, and cytosine, but also for all chloroaqua- and diaqua-platinumated complexes with vitamins studied from the B group. However, the affinities of all analyzed vitamins to platinum derivatives are smaller if compared to guanine (GUA), adenine (ADE), and cytosine (CYT). In Table 1 and Table 2 the energetic characteristics of the complexes with vitamins from the B group and nucleobases with Pt-Chloroaqua are presented, while in Table 3 and Table 4, those with diaqua-platinumated complexes are shown. The results were obtained based on the structures computed at the B3LYP/6-31G(d,p)/LANL2DZ level of theory (Table 1, Table 2 and Table 3). The estimated values of ΔG_r_ were further compared with the results obtained at the MN15/def2-TZVP level of theory (Table 2 and Table 4). Furthermore, the obtained values of ΔG_r_ for Oxaliplatin were compared with the results of Cisplatin [24] and Carboplatin [25].

Oxaliplatin has the best affinity for vitamin B3 (niacin) with value of the Gibbs free energy equal −22.32 kcal/mol (Table 1). It means that niacin easily forms complexes, not only with cis-Pt-Chloroaqua, cis~[Pt(NH_3_)_2_H_2_O]^+^ [24], but also with [PtH_2_OClDACH]^+^. The second highest value of the ΔG*_r_* equal −17.06 kcal/mol was obtained for vitamin B6 (pyridoxal phosphate). For vitamin B1 (thiamine), the ΔG*_r_* value is equal −16.24 kcal/mol in the reaction with lone pair orbitals analogous to N_1_ in purine (Table 1). In the case of reaction, where lone pair orbitals analogous to N_7_ in purine of vitamin B1, the value of affinity is −13.87 kcal/mol (Table 1). The vitamin B2 (riboflavin) has the worst affinity, with slightly negative values of the Gibbs free energy of the reaction, approaching zero (−1.46 kcal/mol). In general, it can be concluded that the order of affinity of Oxaliplatin for B vitamins is similar to Cisplatin [24], and quite different compared with Carboplatin [25] at the B3LYP/6-31G(d,p)/LANL2DZ level of theory.

The results obtained at the MN15/def2-TZVP (Table 2) show a completely different tendency when compared to the results derived from the lower level of theory. At this higher level of calculations, the best affinity to Oxaliplatin was showed by vitamin B1 (thiamine), in reaction with lone pair orbitals analogous to N_1_ in purine, equal −48.91 kcal/mol. Compared to B3LYP/6-31G(d,p)/LANL2DZ level, the vitamin B2 (riboflavin) showed a much greater affinity for the drug, because the ΔG*_r_*is equal −41.55 kcal/mol, at the MN15/def2-TZVP level of theory. At this higher level of calculations, the vitamin B3 (niacin) has the worst affinity, equal −39.37 kcal/mol. 

The energetic characteristics of the Pt-diaqua reactions with derivatives of vitamin B show that the values of ΔG_r_ are more favorable when compared with Pt-Chloroaqua reactions (Table 1 and Table 3), and are found to be in the range from −35.53 kcal/mol to −13.64 kcal/mol (Table 3) at the B3LYP/6-31G(d,p)/LANL2DZ level of theory, and in the range from −50.58 kcal/mol to −40.61 kcal/mol at the MN15/def2-TZVP level of theory.

The affinity of Pt-diaqua reactions with guanine, adenine, and cytosine is higher comparing with Pt-Chloroaqua (Table 3), as could be expected from the previous studies for Cisplatin [24]. As in the case of Pt-Chloroaqua reactions, the highest value of ΔG_r_ is observed for vitamin B3 (niacin), −35.53 kcal/mol. The Pt-diaqua also easily forms bonds with vitamin B6 (pyridoxal phosphate) and vitamin B1 (N_1_, thiamine), which is analogous not only to Pt-Chloroaqua, but also to Cisplatin [25], with values of ΔG_r_ equal −30.60 kcal/mol and −30.15 kcal/mol, respectively (Table 3). The worst affinity is observed for vitamin B2 (riboflavin), −13.64 kcal/mol.

Again, it can be noticed that in the case of Pt-diaqua reactions, the computational results obtained at both levels of theory differ (B3LYP/6-31G(d,p)/LANL2DZ vs MN15/def2-TZVP); for details see Table 3 and Table 4. It was found that, on the basis of the results obtained at the MN15/def2-TZVP level of theory, the best affinity to Oxaliplatin was shown by the vitamin B1 (thiamine) in reaction with lone pair orbitals analogous to N_1_ in purine, and with value of Gibbs Free Energy equal −50.58 kcal/mol. The vitamin B2 (riboflavin) exhibits a much greater affinity for the drug than in the case of application of lower level of theory with value −42.96 kcal/mol (Table 3). The values of the Gibbs free energy of nucleobases are higher compared with the affinity values obtained at the B3LYP/6-31G(d,p)/LANL2DZ (Table 3). It can be concluded that the behavior of Oxaliplatin is similar to Cisplatin in relation to nucleobases (adenine, guanine, and cytosine) and all vitamins from the B group [24] at the B3LYP/6-31G(d, p)/LANL2DZ level of calculations. This level of theory was based on Baik’s studies [35]. However, the Gibbs free energy values were obtained at two levels of theory and compared to each other. The higher level of theory, MN15/def2-TZVP [36,37], was chosen because it describes better possible non-covalent interactions present in the complexes studied. Unfortunately, the use of a higher level of calculations changes the order of the obtained values of the affinity of nucleobases and B vitamins to the tested drug, Oxaliplatin. However, regardless of the level of computation, the order of affinities of the studied vitamins to the drug for the Pt-Chloroaqua and Pt-diaqua derivatives are the same, at the B3LYP/6-31G(d, p)/LANL2DZ level of theory for vitamins B3→B6→B1(N_1_)→B1(N_7_)→B2 and for purines GUA→ADE→CYT, and at the MN15/def2-TZVP level of calculations for vitamins B1(N_1_)→B1(N_7_)→B6→B2→B3 and for purines GUA→CYT→ADE. Regardless of the level of calculations and the forms of the studied structures (chloro- and –diaqua), guanine has the highest affinity for Oxaliplatin. An exception is Pt-Chloroaqua derivatives with the vitamin B1, which showed a greater affinity compared to guanine at the MN15/def2-TZVP level of calculations.

The molar volume was computed for six exemplary complexes with nucleobases to estimate quantitatively changes associated with the replacement of a chlorine atom with a water molecule. The data is presented in Table 5. As is shown, the exchange of the species is associated with conformation changes. This further affects the molar volume of the complexes and their size. Such a study gave us a general overview of possible interactions with macromolecules (e.g., nucleic acid).

**Figure 6:**
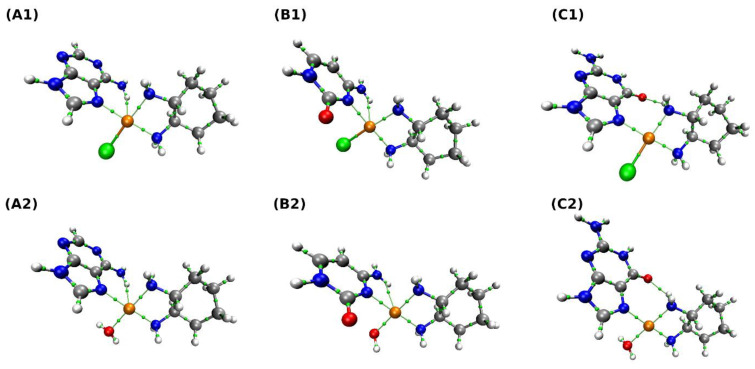
Atoms in molecules (AIM) molecular graphs of the studied complexes. The simulations were performed at the MN15/def2-TZVP level of theory. The BCPs of covalent and non-covalent interactions are presented and marked as small green spheres along bond paths. The designations in Figure are as follows: (**A1**) PtClDACH-Ade, (**A2**) PtH_2_ODACH-Ade, (**B1**) PtClDACH-Cyt, (**B2**) PtH_2_ODACH-Cyt, (**C1**) PtClDACH-Gua, (**C2**) PtH_2_ODACH-Gua.

The results of the AIM analysis are presented in Figure 6 and Table 6. The goal was to investigate topological and electronic structure changes upon the exchange of a chlorine atom with a water molecule. As is shown in Figure 6, the BCPs have been found for covalent and non-covalent interactions. It is worth underlining that, depending on the complex, the network of chemical bonds or non-covalent interactions differs. Therefore, our attention was mostly focused on the Pt interactions with the closest chemical environment. Concerning the complex (A1), it is clear that Pt interacts with the chlorine atom and three nitrogen atoms (one from the adenine ring, two from the NH_2_ groups). There is also a BCP between Pt and a hydrogen atom of the NH_2_ group of adenine. In the case of the complex (A2), the network of interactions is similar. There are BCPs between Pt and nitrogen atoms, the oxygen atom from the water molecule, and the hydrogen atom. Interestingly, there is a BCP between nitrogen and hydrogen atoms from the NH_2_ groups. For PtClDACH-Cyt complex (B1), the following BCPs were found: Pt interacting with a chlorine atom, nitrogen atoms, and a hydrogen atom from the NH_2_ group. The exchange of a chlorine atom with a water molecule (complex (B2)) did not change the binding mode of the Pt atom. However, here, as well as in the case of other complexes, the Cl-Pt bond is characterized by significantly larger electronic density at the BCP than its H_2_O-Pt counterpart. The Pt atom interacts with three nitrogen atoms, an oxygen atom from the water molecule, and with the hydrogen from NH_2_ group. In the complex (C1), BCPs between Pt and chlorine atom and three nitrogen atoms have been observed. There is an intramolecular interaction between the oxygen atom from guanine and the hydrogen atom from the NH_2_ group of Oxaliplatin moiety. In the last analyzed complex (C2), the interactions between Pt and three nitrogen atoms and an oxygen atom from the water molecule were found. There is a BCP between an oxygen atom from guanine and a hydrogen atom from the NH_2_ group of Oxaliplatin moiety. The topological analysis revealed the network of interactions stabilizing the complexes. In addition, it could be useful in the design of new complexes where some of the intramolecular interactions are not preferable. 

In Table 6, the values of electron density and its Laplacian at BCPs are presented. In addition, the potential electronic energy density was calculated to provide a general picture of the strength of the interactions. The data were collected for the BCPs found between Pt and fragments of nucleobases and the Oxaliplatin moiety. Some details of the intramolecular and intermolecular hydrogen bonds are reported. As it is shown, depending on the nucleobase and the presence of the chlorine atom or water molecule in the vicinity of the Pt atom of the Oxaliplatin moiety, the electron density and its Laplacian values were changing. This is also visible in the potential electronic energy density. The values of the electron density and its Laplacian at the BCPs for Pt-Cl and Pt-H_2_O are similar. However, the potential energy density values differ (Table 6).

Time-dependent density functional theory (TD-DFT) was applied to investigate spectroscopic properties of sixteen selected complexes for which experimental UV-Vis measurements were done (see Section 2.2. below). Table S1 presents excitation energies for singlet-singlet transitions in eV as well as in nm. In addition, the oscillator strength is provided. It was found that the most intensive transitions are at 243.56 nm for PtClDACH-Ade, 254.22 nm for PtClDACH-Gua, 250.34 nm for PtClDACH-Cyt, 243.06 nm for PtH_2_ODACH-Ade, 260.50 nm for PtH_2_ODACH-Gua, 250.54 nm for PtH_2_ODACH-Cyt, 252.64 nm for PtClDACH-vitB1(N_1_), 255.04 nm for PtClDACH-vitB1(N_7_), 248.69 nm for PtH_2_ODACH-vitB1(N_1_), 255.04 nm for PtH_2_ODACH-vitB1(N_7_), 443.05 nm for PtClDACH-vitB2, 447.71 nm for PtH_2_ODACH-vitB2, 301.58 nm for PtClDACH-vitB3, 250.47 nm for PtH_2_ODACH-vitB3, 258.68 nm for PtClDACH-vitB6, and 258.55 nm for PtH_2_ODACH-vitB6. The comparison with experimental findings is provided in the end of Section 2.2. 

### 2.2. UV-Vis Experimental Study

**Figure 7:**
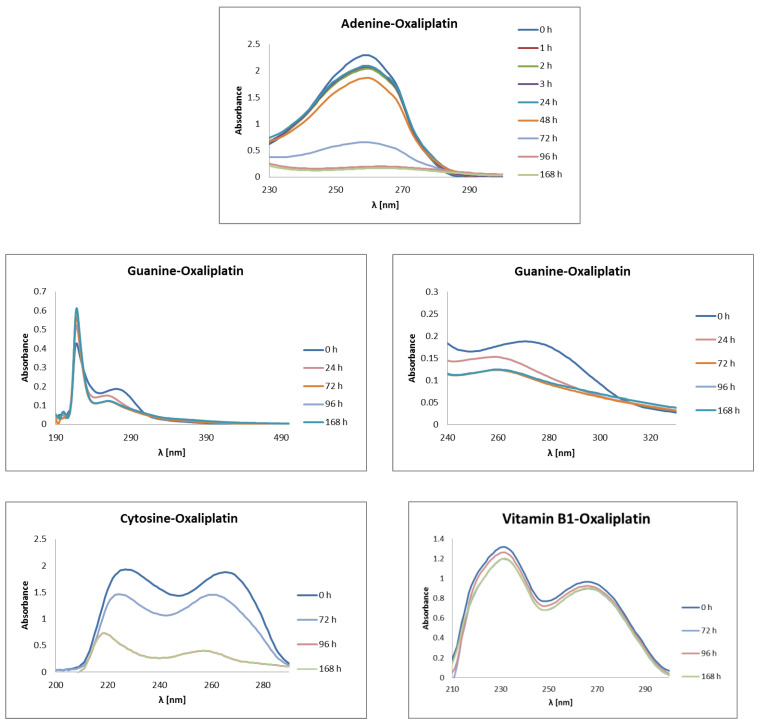

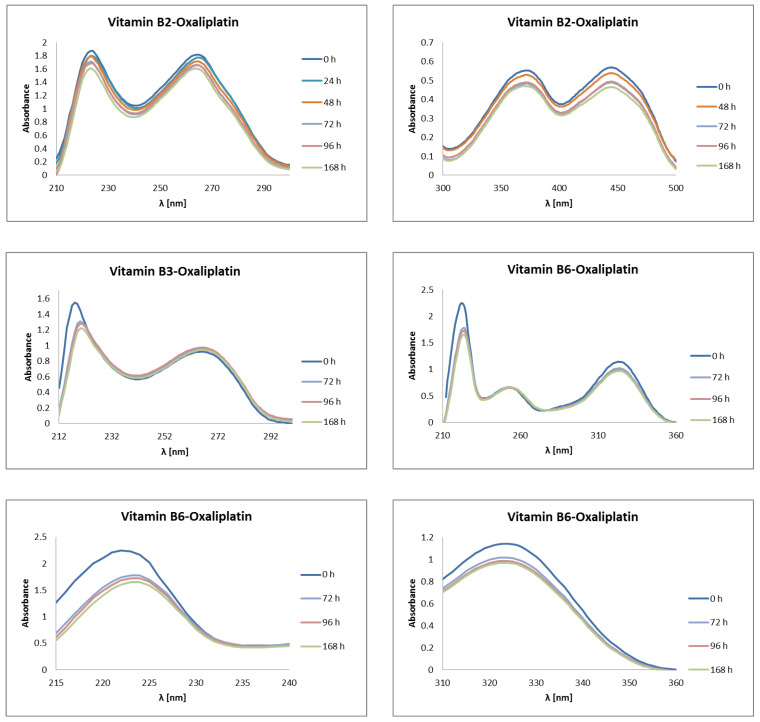
UV-Vis absorption spectra of vitamins from the B group and Oxaliplatin mixture in an incubation buffer (1 mmol L^−1^ phosphate buffer, 4 mmol L^−1^ sodium-chloride, pH 7.4). 0h shows initial concentrations of nucleobases or B vitamins before the Oxaliplatin is added to the mixture. The nucleobases and the B vitamins were incubated at 37 °C with Oxaliplatin in a ratio of 2:1.

Physico-chemical characterization of the interactions of vitamins from the B group and nucleobases with Oxaliplatin were experimentally measured by using the UV-Vis spectroscopic method, in the wavelength range from 190 nm to 500 nm (Figure 7). The UV-Vis measurement was carried out at specific time intervals of 1 h, 2 h, 3 h, 24 h, 48 h, 72 h, 96 h, and 168 h. The mixtures of nucleobases or vitamins and Oxaliplatin (synonyms: [SP-4-2-(1R-trans)]-(1,2-Cyclohexanediamine-N,N′)[ethanedioata(2-)-O,O]platinum; Oxaliplatinum; (SP-4-2)-[(1R,2R)-Cyclohexane-1,2-diamine-κN,κN′]-[ethanedioato(2-)-κO1,κO2]platinum) were incubated in buffer: 1 mmol L^−1^ phosphate buffer, 4 mmol L^−1^ sodiumchloride, pH 7.4. Absorption maxima were determined for all mixtures.

Some of the mixtures showed more than one absorption maximum (Figure 7). The results of the experimental studies confirmed the in silico findings. The mixtures with nucleobases (adenine, guanine, cytosine) showed a significant decrease in the absorbance maximum in relation to the baseline (0 h). In the case of complexation of Oxaliplatin with adenine, where maximum absorption was observed at λ = 260 nm, a significant decrease in baseline is observed after 72 h, approximately 3.5 times the initial concentration of adenine in the mixture, and over 14 times after 168 h. Slight decrease in the concentration of adenine in the mixture, one time, is observed after 1 h, 2 h, 3 h. After 48 h, the concentration of adenine in the mixture is reduced twofold from the initial concentration. The decrease in the absorbance of baseline is caused by the formation of adenine-Oxaliplatin complexes. Guanine has two absorbance maxima, at λ = 218 nm and λ = 270 nm. Only the latter one decreases after the complexation of guanine with Oxaliplatin (Figure 7). After the complexation of guanine with Oxaliplatin, a shift in the absorbance maximum of guanine towards shorter wavelengths, λ = 260 nm, was noticed. First, a significant drop in baseline was observed after 24 h. Even so, the greatest decrease in the absorbance maximum, of about 1.5 times, occurred after 72 h. Extending the incubation time of the mixture of guanine with Oxaliplatin did not lead to any further changes in the amount of the absorbance peak. Cytosine showed two maxima of absorption, at λ = 227 nm and λ = 266 nm. Both of these significantly decreased after 168 h: by 2.7 times and 4.5 times, respectively. After 72 h, the complexation of cytosine–Oxaliplatin causes a decrease in the initial concentration of this nucleobase in the mixture by only onefold. It was observed that the time range 1–3 h is not enough for complexation in this case. The complexation of Oxaliplatin with B vitamins is much slower than that of nucleobases. Up to 3 h, the decrease in concentrations of vitamins in the mixture are insignificant. Meaningful drops in the concentration of vitamin B in the mixture, greater than onefold each time, are observed after 72 h and 168 h. The pyridoxal phosphate (vitamin B6) interacts best with the chemotherapeutic, among all vitamins studied. In this case, after 168 h a decrease in the concentration of about 1.5 times is observed. In the case of the vitamins B1 (thiamine) and B2 (riboflavin), after 72 h a decrease of greater than 1 time is observed in the baseline and, respectively, after a little more after 168 h. In the case of vitamins B3 (niacin) and B6 (pyridoxal phosphate), a significant decrease in the baseline is visible after 96 h, while after 168 h there is no further reduction in the concentration of the tested vitamins. The size of the reduction in the concentration of the tested vitamins depends little on the wavelength at which the maximum absorbance occurs.

Let us compare the theoretical and experimental excitation energies for the selected complexes. The theoretical findings concerning the most intensive singlet-singlet transitions for PtClDACH-Ade and PtH_2_ODACH-Ade are 243.56 nm and 243.06 nm, respectively. The experimental data showed that the most intensive band occurred at 260 nm. Concerning the complex with guanine, the computed values are 254.22 nm for PtClDACH-Gua and 260.50 nm for PtH_2_ODACH-Gua, while the experimental spectra showed two absorbance maxima, at 218 nm and 270 nm, respectively. The last nucleic base taken into consideration in the study was cytosine. For the complexes with a chlorine atom and water molecule, the most intensive transitions were noticed at 250.34 nm (for PtClDACH-Cyt) and 250.54 nm (for PtH_2_ODACH-Cyt), whereas the experimental findings showed two absorption maxima, at 227 nm and 266 nm. The computed values for vitamin B1 are as follows: 252.64 nm for PtClDACH-vit. B1(N_1_), 248.69 nm for PtH_2_ODACH-vit. B1(N_1_), 255.04 nm for PtClDACH-vit. B1(N_7_), and 255.04 nm for PtH_2_ODACH-vit. B1(N_7_), while the experimental data showed the absorption maximum at 231 nm and 266 nm, respectively. For the complexes with vitamin B2, we obtained 443.05 nm for PtClDACH-vitB2 and 447.71 nm for PtH_2_ODACH-vitB2, based on TD-DFT, which is in a good agreement with experimental data locating the maximum absorbance band at 445 nm (as well as at 221 nm, 266 nm, and 372 nm). Concerning vitamin B3, the computational results showed the most intensive singlet-singlet transitions at 301.58 nm for PtClDACH-vitB3 and 250.47 nm for PtH_2_ODACH-vitB3. In the experimental spectrum, the absorption maximum was found at 218 nm and 266 nm. Finally, we have computed absorption spectra for vitamin B6. It was found that the absorbance maxima occurred at 258.68 nm for PtClDACH-vitB6 and at 258.55 nm for PtH_2_ODACH-vitB6. The experimental spectrum showed the absorbance maximum at 222 nm, 252 nm, and 323 nm. The computed UV-Vis spectra are shown in Figure S1. The comparison showed a very good agreement between that computed at the MN15/def2-TZVP level of theory with application of the Polarizable Continuum Model (PCM) and water as a solvent with experimental spectra. 

## 3. Materials and Methods

Since B vitamins are similar in structure to nucleobases, possessing aromatic rings with the nitrogen atom N_7_/N_1_, they could easily react with Oxaliplatin (as has been shown in earlier studies of Cisplatin) [24]. Such reaction could decrease the therapeutic effect of the anticancer drug. This is the main reason for the chemical affinity studies of Oxaliplatin to vitamins from the B group. Two possible reactions were investigated in which monoaqua or diaqua complexes play an electrophile role [38], namely [PtH_2_OClDACH]^+^ and [Pt(H_2_O)_2_DACH]^2+^. In this respect, theoretical and experimental studies were performed.

### 3.1. Theoretical Study

Density functional theory (DFT) [39,40] was applied to develop theoretical models describing the geometric, energetic, and electronic structure parameters of the compounds studied. The models for quantum-chemical simulations were prepared manually using the Molden program [41]. The energy minimization was carried out at the B3LYP/6-31G(d, p) [42] and MN15/def2-TZVP [36,37] levels of theory. However, the atomic orbitals of the platinum atom were represented by the LANL2DZ basis set, which includes relativistic effective core potentials needed in the case of heavy atoms concerning the simulations with application of the B3LYP functional [43]. Such methodology was applied by Baik and co-workers [35]. The quantum-chemical computations were performed using the Gaussian 09 Rev. A.02 [44] and Gaussian 16 Rev. C.01 [45,46] suite of programs. The harmonic vibrational frequencies calculations were carried out to confirm that the obtained structures correspond with minima on the potential energy surface (PES) as well as to derive the zero-point energy (ZPE). The same level of theory was used in the case of vibrational entropy corrections at room temperature. A self-consistent reaction field (SCRF) approach was applied to reproduce the polar environment influence and to estimate the solvation free energies [47,48]. The polarizable continuum model (IEF-PCM formulation) was used to describe the aqueous solution with a dielectric constant of 78 for water [49]. By adding thermal corrections to the enthalpy and entropy terms, contributions of ZPE corrections and continuum solvation free energies, the chemical affinity was computed. Finally, for six selected complexes (denoted as: PtClDACH-Ade, PtH_2_ODACH-Ade, PtClDACH-Cyt, PtH_2_ODACH-Cyt, PtClDACH-Gua, PtH_2_ODACH-Gua) molar volume was computed, and the electronic structure was analyzed on the basis of atoms in molecules (AIM) theory [50]. The electron density and its Laplacian were estimated at bond critical points (BCPs) and the potential electronic energy density (V_r_) was computed. The AIM analysis was carried out with assistance of the Multiwfn 3.7 program [51]. Finally, time-dependent density functional theory (TD-DFT) [47] was applied for selected complexes investigated experimentally (for more details see the text below). We have applied the same computational setup as described above (MN15/def2-TZVP, PCM) concerning the DFT calculations. We have carried out calculations of the vertical excitation energies from the ground state (S_0_) to the first five excited states (S_1_, S_2_, S_3_, S_4_, and S_5_) for the optimized complexes. The TD-DFT study was performed with the Gaussian 16 Rev. C.01 [45,46] suite of programs. The graphical presentation of the obtained results was prepared with the VMD 1.9.3 program [52]. 

### 3.2. Experimental Study (UV-Vis Measurements)

The spectrophotometrical measurements were performed on a Biosens UV-6000 spectrophotometer (Biosens, Warsaw, Poland). Oxaliplatin (synonims: [SP-4-2-(1R-trans)]-(1,2-Cyclohexanediamine-N,N′)[ethanedioata(2--)-O,O]platinum; Oxaliplatinum; (SP-4-2)-[(1R,2R)-Cyclohexane-1,2-diamine-κN,κN′]-[ethanedioato(2-)-κO1,κO2]platinum), and vitamins from B group were dissolved in the incubation buffer (1 mmol L^–1^ phosphate buffer, 4 mmol L^–1^ sodiumchloride, pH 7.4). During the measurements, of the nucleobases and the vitamins from B group were incubated at 37 °C with Oxaliplatin in a ratio of 2:1. The initial concentrations of study structures are 0.352 mmol L^–1^ for adenine, 0.614 mmol L^–1^ for cytosine, 0.503 mmol L^–1^ for guanine, 0.178 mmol L^–1^ for vitamin B1 (thiamine), 0.107 mmol L^–1^ for vitamin B2 (riboflavin), 0.712 mmol L^–1^ for vitamin B3 (niacin), and 0.365 mmol L^–1^ for vitamin B6 (pyridoxal phosphate). Physico-chemical characteristics of the interaction of nucleobases (adenine, guanine, cytosine) or vitamins from B group with Oxaliplatin was performed by using UV-Vis spectroscopic technique, in wavelength range from 190 nm to 500 nm. Depending on the type of nucleobases and vitamins, the tested compounds showed maximum absorptions at different wavelengths (Figure 7 and Figure S2). The cytosine and vitamins from B group showed more than one absorption maximum (Figure 7 and Figure S2). The samples of mixture of nucleobases or vitamins B and Oxaliplatin were taken after 0 h, 1 h, 2 h, 3 h, 24 h, 72 h, and 168 h incubation. 

## 4. Conclusions

Theoretical studies based on DFT, TD-DFT, and experimental (UV-Vis) investigations were carried out on complexes formed with nucleobases/vitamins from the B group with Oxaliplatin. The affinity of the complexes was estimated to shed more light on the interactions and their consequences on cancer therapy. The order of affinity of Oxaliplatin for B vitamins for both monoaqua [PtH_2_OClDACH]^+^ and diaqua [Pt(H_2_O)_2_DACH]^2+^ derivatives is niacin (vitamin B3), pyridoxal phosphate (vitamin B6), thiamine (vitamin B1), and riboflavin (vitamin B2), as obtained at the B3LYP/6-31G(d,p) level of theory. However, in the case of the MN15/def2-TZVP level of theory, the order of the affinity of Oxaliplatin for the vitamins studied was different: from thiamine (vitamin B1), to pyridoxal phosphate (vitamin B6), riboflavin (vitamin B2), and up to niacin (vitamin B3). Theoretical studies confirmed the clinical observations and suggest high therapeutic effectiveness of Oxaliplatin in cancer treatment. The AIM theory application revealed the existence of intramolecular non-covalent interactions, which could stabilize the conformation and further provided an impact on the interactions with macromolecules. Qualitative agreement between the computed and experimental absorption spectra was observed. The UV-Vis measurements confirmed the findings obtained based on theoretical approaches. A significant decrease of the concentrations of nucleobases in the mixtures were observed after the complexation with Oxaliplatin. It was noted that the vitamins from the B group form weaker and more time-consuming complexes with the chemotherapeutic studied. It can be stated that the B vitamins from the juice the patients drink are less likely to interfere with cancer treatment involving Oxaliplatin, based on the data presented in the study.

## Figures and Tables

**Table 1:** The energetic characteristics of the Pt-Chloroaqua reactions with vitamin B and nucleobases obtained at the B3LYP/6-31G(d,p)/LANL2DZ level of theory. All energies are given in kcal/mol. Symbol Pt* stands for PtClDACH.

Number of Reaction	ΔG_r_	Reaction
1	−13.87	[Pt*]^+^ + [B1_(N7)_]^+^ → [Pt*-B1_(N7)_]^2+^
2	−16.24	[Pt*]^+^ + [B1_(N1)_]^+^ → [Pt*-B1_(N1)_]^2+^
3	−1.46	[Pt*]^+^ + B2 → [Pt*-B2]^+^
4	−22.32	[Pt*]^+^ + B3 → [Pt*-B3]^+^
5	−17.06	[Pt*]^+^ + B6 → [Pt*-B6]^+^
6	−33.46	[Pt*]^+^ + GUA → [Pt*-GUA]^+^
7	−28.80	[Pt*]^+^ + ADE → [Pt*-ADE]^+^
8	−26.89	[Pt*]^+^ + CYT → [Pt*-CYT]^+^

**Table 2:** The energetic characteristics of the Pt-Chloroaqua reactions with vitamin B and nucleobases obtained at the MN15/def2-TZVP level of theory. All energies are given in kcal/mol. Symbol Pt* stands for PtClDACH.

Number of Reaction	ΔG_r_	Reaction
1	−46.48	[Pt*]^+^ + [B1_(N7)_]^+^ → [Pt*-B1_(N7)_]^2+^
2	−48.91	[Pt*]^+^ + [B1_(N1)_]^+^ → [Pt*-B1_(N1)_]^2+^
3	−41.55	[Pt*]^+^ + B2 → [Pt*-B2]^+^
4	−39.37	[Pt*]^+^ + B3 → [Pt*-B3]^+^
5	−45.93	[Pt*]^+^ + B6 → [Pt*-B6]^+^
6	−46.10	[Pt*]^+^ + GUA → [Pt*-GUA]^+^
7	−42.59	[Pt*]^+^ + ADE → [Pt*-ADE]^+^
8	−45.23	[Pt*]^+^ + CYT → [Pt*-CYT]^+^

**Table 3:** The energetic characteristics of the Pt-diaqua reactions with vitamin B and nucleobases obtained at the B3LYP/6-31G(d,p)/LANL2DZ level of theory. All energies are given in kcal/mol.

Number of Reaction	ΔG_r_	Reaction
1	−25.10	[Pt**]^2+^ + [B1_(N7)_]^+^ → [Pt**-B1_(N7)_]^3+^
2	−30.15	[Pt**]^2+^ + [B1_(N1)_]^+^ → [Pt**-B1_(N1)_]^3+^
3	−13.64	[Pt**]^2+^ + B2 → [Pt**-B2]^2+^
4	−35.53	[Pt**]^2+^ + B3 → [Pt**-B3]^2+^
5	−30.60	[Pt**]^2+^ + B6 → [Pt**-B6]^2+^
6	−50.03	[Pt**]^2+^ + GUA → [Pt**-GUA]^2+^
7	−41.37	[Pt**]^2+^ + ADE → [Pt**-ADE]^2+^
8	−29.07	[Pt**]^2+^ + CYT → [Pt**-CYT]^2+^

where Pt** symbol is PtH_2_ODACH.

**Table 4:** The energetic characteristics of the Pt-diaqua reactions with vitamin B and nucleobases obtained at the MN15/def2-TZVP level of theory. All energies are given in kcal/mol.

Number of Reaction	ΔG_r_	Reaction
1	−46.45	[Pt**]^2+^ + [B1_(N7)_]^+^ → [Pt**-B1_(N7)_]^3+^
2	−50.58	[Pt**]^2+^ + [B1_(N1)_]^+^ → [Pt**-B1_(N1)_]^3+^
3	−42.96	[Pt**]^2+^ + B2 → [Pt**-B2]^2+^
4	−40.61	[Pt**]^2+^ + B3 → [Pt**-B3]^2+^
5	−47.11	[Pt**]^2+^ + B6 → [Pt**-B6]^2+^
6	−51.01	[Pt**]^2+^ + GUA → [Pt**-GUA]^2+^
7	−43.67	[Pt**]^2+^ + ADE → [Pt**-ADE]^2+^
8	−47.89	[Pt**]^2+^ + CYT → [Pt**-CYT]^2+^

where Pt** symbol is PtH_2_ODACH.

**Table 5:** Molar volume (cm^3^/mol) of selected complexes computed at the MN15/def2-TZVP level of theory. Molecular structures of the complexes are shown in Figure 6.

**Volume**	**PtClDACH-Ade** **(A1)**	**PtH2ODACH-Ade** **(A2)**	**PtClDACH-Cyt** **(B1)**	**PtH2ODACH-Cyt** **(B2)**	**PtClDACH-Gua** **(C1)**	**PtH2ODACH-Gua** **(C2)**
	205.392	183.606	188.870	181.336	198.370	210.671

**Table 6:** Bond Critical Points (BCPs) and potential energy density obtained at the MN15/def2-TZVP level of theory for six selected complexes. Electron density ρ_BCP_ is given in e_*_a_0_^−3^ atomic units, and its Laplacian ∇^2^ρ_BCP_ in e_*_a_0_^−5^ units.

BCP	PtClDACH-Ade (A1)			BCP	PtH_2_ODACH-Ade (A2)		
	ρ_BCP_	∇^2^ρ_BCP_	V(r)		ρ_BCP_	∇^2^ρ_BCP_	V(r)
H_2_N-Pt	1.23 × 10^−1^	4.16 × 10^−1^	−1.94 × 10^−1^	H_2_O-Pt	9.63 × 10^−2^	4.65 × 10^−1^	−1.66 × 10^−1^
N-Pt	1.27 × 10^−1^	4.41 × 10^−1^	−2.06 × 10^−1^	H_2_N-Pt	1.39 × 10^−1^	3.89 × 10^−1^	−2.14 × 10^−1^
NH-Pt	1.39 × 10^−2^	3.93 × 10^−2^	−7.94 × 10^−3^	N-Pt	1.29 × 10^−1^	4.16 × 10^−1^	−2.06 × 10^−1^
H_2_N-Pt	1.26 × 10^−1^	4.15 × 10^−1^	−2.00 × 10^−1^	H_2_N-Pt	1.26 × 10^−1^	3.99 × 10^−1^	−1.97 × 10^−1^
Cl-Pt	1.04 × 10^−1^	1.89 × 10^−1^	−1.28 × 10^−1^	NH-Pt	1.38 × 10^−2^	4.00 × 10^−2^	−8.12 × 10^−3^
**BCP**	**PtClDACH-Cyt (B1)**			**BCP**	**PtH_2_ODACH-Cyt (B2)**		
	**ρ_BCP_**	**∇^2^ρ_BCP_**	**V(r)**		**ρ_BCP_**	**∇^2^ρ_BCP_**	**V(r)**
Cl-Pt	1.02 × 10^−1^	1.85 × 10^−1^	−1.25 × 10^−1^	H_2_O-Pt	9.61 × 10^−2^	4.59 × 10^−1^	−1.65 × 10^−1^
NH-Pt	1.88 × 10^−2^	6.01 × 10^−2^	−1.32 × 10^−2^	H_2_N-Pt	1.38 × 10^−1^	3.95 × 10^−1^	−2.14 × 10^−1^
H_2_N-Pt	1.23 × 10^−1^	4.24 × 10^−1^	−1.97 × 10^−1^	N-Pt	1.24 × 10^−1^	3.88 × 10^−1^	−1.92 × 10^−1^
N-Pt	1.21 × 10^−1^	4.12 × 10^−1^	−1.92 × 10^−1^	H_2_N-Pt	1.26 × 10^−1^	4.03 × 10^−1^	−1.98 × 10^−1^
H_2_N-Pt	1.25 × 10^−1^	4.14 × 10^−1^	−1.98 × 10^−1^	NH-Pt	1.74 × 10^−2^	5.83 × 10^−2^	−1.24 × 10^−2^
**BCP**	**PtClDACH-Gua (C1)**			**BCP**	**PtH_2_ODACH-Gua (C2)**		
	**ρ_BCP_**	**∇^2^ρ_BCP_**	**V(r)**		**ρ_BCP_**	**∇^2^ρ_BCP_**	**V(r)**
Cl-Pt	1.02 × 10^−1^	1.97 × 10^−1^	−1.27 × 10^−1^	H_2_O-Pt	9.54 × 10^−2^	4.69 × 10^−1^	−1.65 × 10^−1^
N-Pt	1.25 × 10^−1^	4.53 × 10^−1^	−2.06 × 10^−1^	N-Pt	1.28 × 10^−1^	4.31 × 10^−1^	−2.07 × 10^−1^
H_2_N-Pt	1.25 × 10^−1^	4.12 × 10^−1^	−1.96 × 10^−1^	H_2_N-Pt	1.39 × 10^−1^	3.77 × 10^−1^	−2.12 × 10^−1^
H_2_N-Pt	1.26 × 10^−1^	4.15 × 10^−1^	−1.99 × 10^−1^	H_2_N-Pt	1.27 × 10^−1^	4.04 × 10^−1^	−1.99 × 10^−1^
O...HN	1.84 × 10^−2^	7.27 × 10^−2^	−1.23 × 10^−2^	O...HN	1.90 × 10^−2^	7.36 × 10^−2^	−1.27 × 10^−2^

## Data Availability

The data presented in the current study are available in the article and in the associated Supplementary Material.

## Supplementary Materials

The following supporting information can be downloaded at: https://www.mdpi.com/article/10.3390/ijms231810567/s1.

## References

[1] Seetharam R.N. (2010). Oxaliplatin: Preclinical perspectives on the mechanisms of action, response and resistance. Ecancermedicalscience.

[2] Martinez-Balibrea E., Martínez-Cardús A., Ginés A., Ruiz de Porras V., Moutinho C., Layos L., Manzano J.L., Bugés C., Bystrup S., Esteller M. (2015). Tumor-Related Molecular Mechanisms of Oxaliplatin Resistance. Mol. Cancer Ther..

[3] Cvitkovic E. (1998). Ongoing and unsaid on oxaliplatin: The hope. Br. J. Cancer.

[4] Wang D., Lippard S.J. (2005). Cellular processing of platinum anticancer drugs. Nat. Rev. Drug Discov..

[5] Di Francesco A.M., Ruggiero A., Riccardi R. (2002). Cellular and molecular aspects of drugs of the future: Oxaliplatin. Cell. Mol. Life Sci..

[6] Rivory L.P. (2002). Experimental and Clinical Pharmacology: New drugs for colorectal cancer-mechanisms of action. Aust. Prescr..

[7] Farrell N.P. (2004). Preclinical perspectives on the use of platinum compounds in cancer chemotherapy. Semin. Oncol..

[8] Sharma S., Gong P., Temple B., Bhattacharyya D., Dokholyan N.V., Chaney S.G. (2007). Molecular dynamic simulations of cisplatin- and oxaliplatin-d(GG) intrastand cross-links reveal differences in their conformational dynamics. J. Mol. Biol..

[9] Chaney S.G., Campbell S.L., Bassett E., Wu Y. (2005). Recognition and processing of cisplatin- and oxaliplatin-DNA adducts. Crit. Rev. Oncol. Hematol..

[10] Raymond E., Chaney S.G., Taamma A., Cvitkovic E. (1998). Oxaliplatin: A review of preclinical and clinical studies. Ann. Oncol..

[11] Rixe O., Ortuzar W., Alvarez M., Parker R., Reed E., Paull K., Fojo T. (1996). Oxaliplatin, tetraplatin, cisplatin, and carboplatin: Spectrum of activity in drug-resistant cell lines and in the cell lines of the national cancer institute’s anticancer drug screen panel. Biochem. Pharmacol..

[12] Martin L.P., Hamilton T.C., Schilder R.J. (2008). Platinum resistance: The role of DNA repair pathways. Clin. Cancer Res..

[13] Meyerhardt J.A., Mayer R.J. (2005). Systemic therapy for colorectal cancer. N. Engl. J. Med..

[14] Webster R.G., Brain K.L., Wilson R.H., Grem J.L., Vincent A. (2005). Oxaliplatin induces hyperexcitability at motor and autonomic neuromuscular junctions through effects on voltage-gated sodium channels. Br. J. Pharmacol..

[15] Gebremedhn E.G., Shortland P.J., Mahns D.A. (2018). The incidence of acute oxaliplatin-induced neuropathy and its impact on treatment in the first cycle: A systematic review. BMC Cancer.

[16] Fukuda M., Ohe Y., Kanzawa F., Oka M., Hara K., Saijo N. (1995). Evaluation of novel platinum complexes, inhibitors of topoisomerase I and II in non-small cell lung cancer (NSCLC) sublines resistant to cisplatin. Anticancer Res..

[17] Kidani Y., Inagaki K., Saito R., Tsukagoshi S. (1976). Synthesis and anti tumor activities of platinum(II) complexes of 1,2 diaminocyclohexane isomers and their related derivatives. Wadley Med. Bull..

[18] Göschl S., Schreiber-Brynzak E., Pichler V., Cseh K., Heffeter P., Jungwirth U., Jakupec M.A., Berger W., Keppler B.K. (2017). Comparative studies of oxaliplatin-based platinum(iv) complexes in different in vitro and in vivo tumor models. Metallomics.

[19] Riccardi A., Ferlini C., Meco D., Mastrangelo R., Scambia G., Riccardi R. (1999). Antitumour activity of oxaliplatin in neuroblastoma cell lines. Eur. J. Cancer.

[20] Dunn T.A., Schmoll H.J., Grünwald V., Bokemeyer C., Casper J. (1997). Comparative cytotoxicity of oxaliplatin and cisplatin in non-seminomatous germ cell cancer cell lines. Investig. New Drugs.

[21] Pendyala L., Creaven P.J. (1993). In Vitro Cytotoxicity, Protein Binding, Red Blood Cell Partitioning, and Biotransformation of Oxaliplatin. Cancer Res..

[22] Kraker A.J., Moore C.W. (1988). Accumulation of cis-Diamminedichloroplatinum(II) and Platinum Analogues by Platinum-resistant Murine Leukemia Cells in Vitro. Cancer Res..

[23] Monti D.M., Loreto D., Iacobucci I., Ferraro G., Pratesi A., D’Elia L., Monti M., Merlino A. (2022). Protein-based delivery systems for anticancer metallodrugs: Structure and biological activity of the oxaliplatin/β-lactoglobulin adduct. Pharmaceuticals.

[24] Szefler B., Czeleń P., Szczepanik A., Cysewski P. (2019). Does the Affinity of Cisplatin to B-Vitamins Impair the Therapeutic Effect in the Case of Patients with Lung Cancer-consuming Carrot or Beet Juice?. Anticancer Agents Med. Chem..

[25] Szefler B., Czeleń P., Krawczyk P. (2021). The Affinity of Carboplatin to B-Vitamins and Nucleobases. Int. J. Mol. Sci..

[26] Hodgkinson E., Neville-Webbe H.L., Coleman R.E. (2006). Magnesium Depletion in Patients Receiving Cisplatin-based Chemotherapy. Clin. Oncol..

[27] Fischer J., Robin Ganellin C. (2006). Analogue-based Drug Discovery. Chem. Int.—Newsmag. IUPAC.

[28] Lockwood G.F., Greenslade D., Brienza S., Bayssas M., Gamelin E., American Association for Cancer Research (1995). Clinical Cancer Research: An Official Journal of the American Association for Cancer Research.

[29] Puisset F., Schmitt A., Chatelut E. (2014). Standardization of Chemotherapy and Individual Dosing of Platinum Compounds. Anticancer Res..

[30] Kurter H., Yeşil J., Daskin E., Çalibaşi Koçal G., Ellidokuz H., Başbinar Y. (2021). Drug Resistance Mechanisms on Colorectal Cancer. J. Basic Clin. Health Sci..

[31] Han C.H., Khwaounjoo P., Hill A.G., Miskelly G.M., McKeage M.J. (2017). Predicting effects on oxaliplatin clearance: In vitro, kinetic and clinical studies of calcium- and magnesium-mediated oxaliplatin degradation. Sci. Rep..

[32] Graham M.A., Lockwood G.F., Greenslade D., Brienza S., Bayssas M., Gamelin E. (2000). Clinical Pharmacokinetics of Oxaliplatin: A Critical Review. Clin. Cancer Res..

[33] Alcindor T., Beauger N. (2011). Oxaliplatin: A review in the era of molecularly targeted therapy. Curr. Oncol..

[34] World Cancer Research Fund/American Institute for Cancer (2018). Diet, nutrition, physical activity and breast cancer. Contin. Updat. Proj. Expert Rep..

[35] Baik M.-H., Friesner R.A., Lippard S.J. (2003). Theoretical Study of Cisplatin Binding to Purine Bases: Why Does Cisplatin Prefer Guanine over Adenine?. J. Am. Chem. Soc..

[36] Yu H.S., He X., Li S.L., Truhlar D.G. (2016). MN15: A Kohn–Sham global-hybrid exchange–correlation density functional with broad accuracy for multi-reference and single-reference systems and noncovalent interactions. Chem. Sci..

[37] Weigend F., Ahlrichs R. (2005). Balanced basis sets of split valence, triple zeta valence and quadruple zeta valence quality for H to Rn: Design and assessment of accuracy. Phys. Chem. Chem. Phys..

[38] Legendre F., Bas V., Kozelka J., Chottard J.C. (2000). A complete kinetic study of GG versus AG platination suggests that the doubly aquated derivatives of cisplatin are the actual DNA binding species. Chem.-A Eur. J..

[39] Kohn W., Sham L.J. (1965). Self-consistent equations including exchange and correlation effects. Phys. Rev..

[40] Hohenberg P., Kohn W. (1964). Inhomogeneous Electron Gas. Phys. Rev..

[41] Schaftenaar G., Noordik J.H. (2000). Molden: A pre- and post-processing program for molecular and electronic structures *. J. Comput. Aided. Mol. Des..

[42] Becke A.D. (1993). Densityfunctional thermochemistry. III. The role of exact exchange Density-functional thermochemistry. III. The role of exact exchange. J. Chem. Phys. Addit. Inf. J. Chem. Phys. J. Homepage.

[43] Hay P.J., Wadt W.R. (1985). Ab initio effective core potentials for molecular calculations. Potentials for the transition metal atoms Sc to Hg. J. Chem. Phys..

[44] Frisch M.J., Trucks G.W., Schlegel H.B., Scuseria G.E., Robb M.A., Cheeseman J.R., Scalmani G., Barone V., Mennucci B., Petersson G.A. Gaussian 09 Citation|Gaussian.com. http://gaussian.com/g09citation/.

[45] Ortiz Quantum Chemistry Group: Collaboration with Gaussian Inc. https://www.auburn.edu/cosam/faculty/chemistry/ortiz/research/info_gaussian.html.

[46] Gaussian 16|Gaussian.com. https://gaussian.com/gaussian16/.

[47] Cramer C.J., Truhlar D.G. (1999). Implicit Solvation Models: Equilibria, Structure, Spectra, and Dynamics. Chem. Rev..

[48] Marten B., Kim K., Cortis C., Friesner R.A., Murphy R.B., Ringnalda M.N., Sitkoff D., Honig B. (1996). New Model for Calculation of Solvation Free Energies: Correction of Self-Consistent Reaction Field Continuum Dielectric Theory for Short-Range Hydrogen-Bonding Effects. J. Phys. Chem..

[49] Barone V., Cossi M., Tomasi J. (1998). A new definition of cavities for the computation of solvation free energies by the polarizable continuum model. J. Chem. Phys..

[50] Bader R.F.W. (1990). Atoms in Molecules: A Quantum Theory.

[51] Lu T., Chen F. (2012). Multiwfn: A multifunctional wavefunction analyzer. J. Comput. Chem..

[52] Humphrey W., Dalke A., Schulten K. (1996). VMD: Visual molecular dynamics. J. Mol. Graph..

