# Supplementary material for: Affinities to Oxaliplatin: Vitamins from B Group vs. Nucleobases
Source: Int J Mol Sci. 2022 Sep 12;23(18):10567. doi: 10.3390/ijms231810567 (PMC9503415; doi:10.3390/ijms231810567)
Supplement: Supplementary file 1 [file ijms-23-10567-s001.zip › ijms-1882000-supplementary.pdf]

Supplementary Material

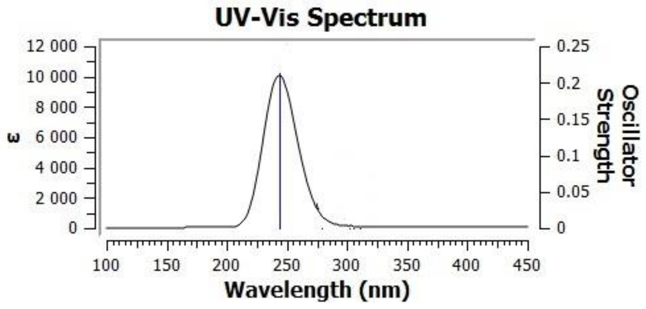

PtClDACH-Ade

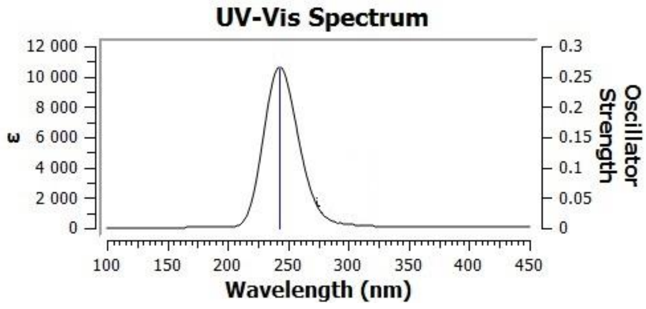

PtH$_2$ODACH-Ade

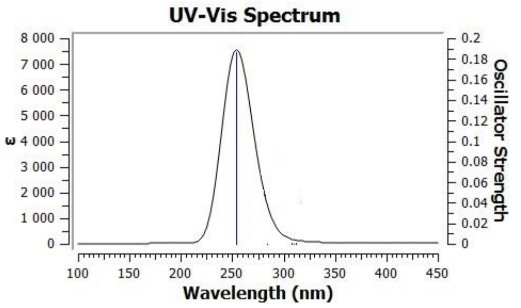

PtClDACH-Gua

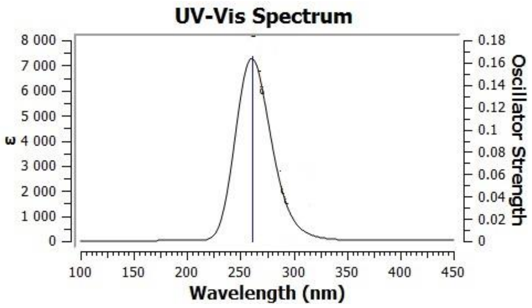

PtH$_2$ODACH-Gua

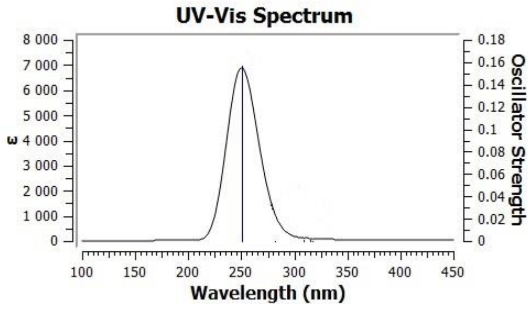

PtClDACH-Cyt

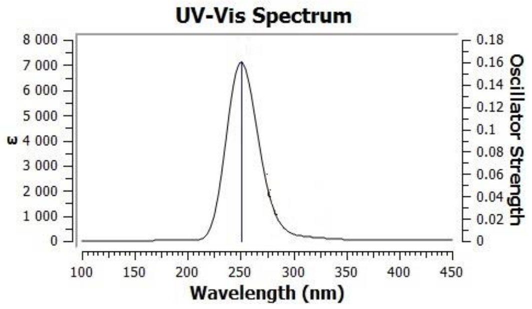

PtH$_2$ODACH-Cyt

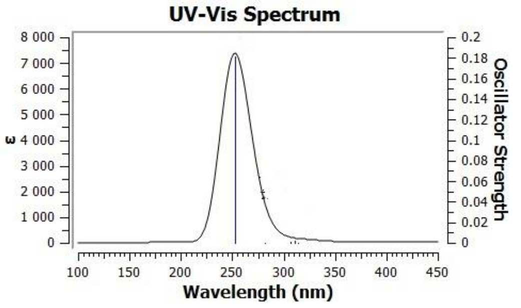

PtClDACH-B1(N$_1$)

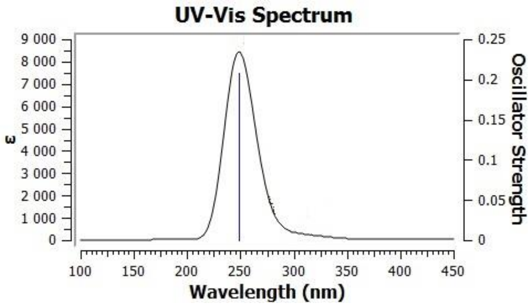

PtH$_2$ODACH-B1(N$_1$)

Figure S1. Computed (MN15/def2-TZVP level of theory with PCM model and water as a solvent) UV-Vis spectra for selected Oxaliplatin complexes with nucleobases and B vitamins.

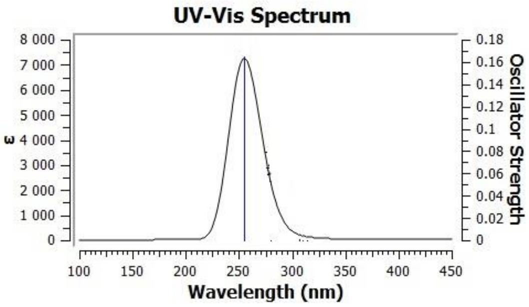

PtClDACH-vitB1(N_7)

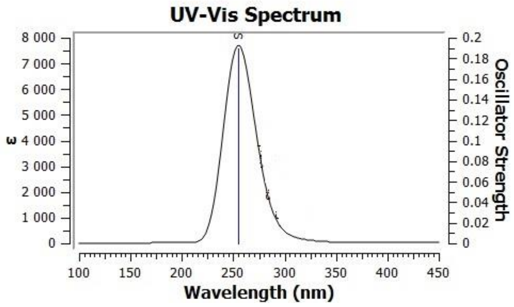

PtH_2ODACH-vitB1(N_7)

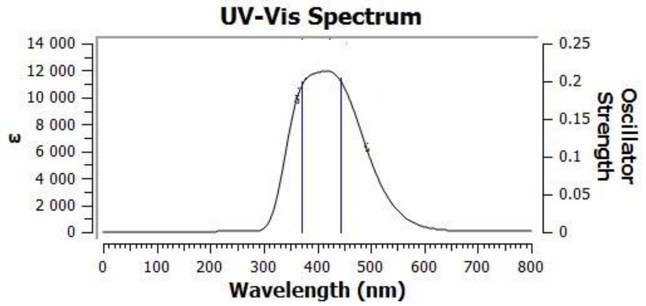

PtClDACH-vitB2

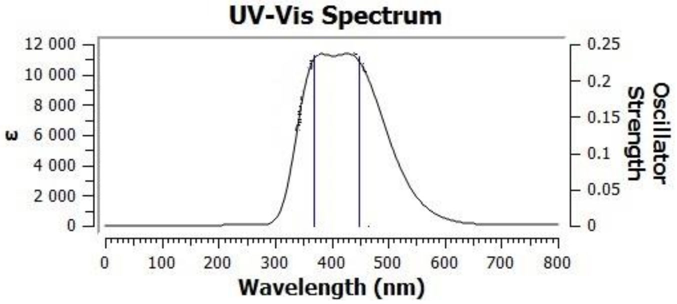

PtH_2ODACH-vitB2

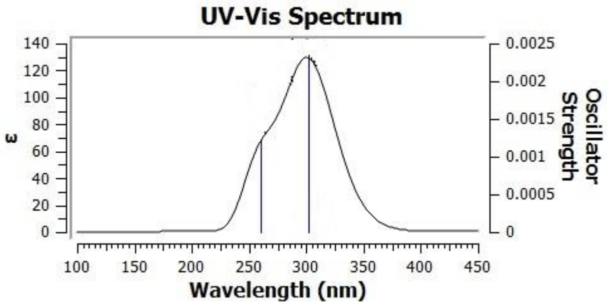

PtClDACH-vitB3

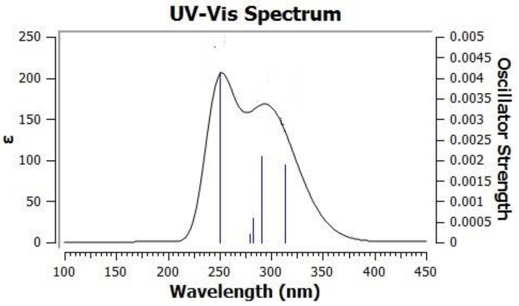

PtH_2ODACH-vitB3

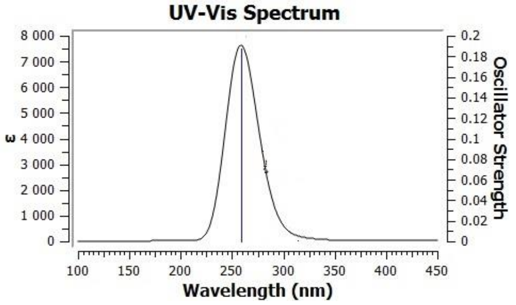

PtClDACH-vitB6

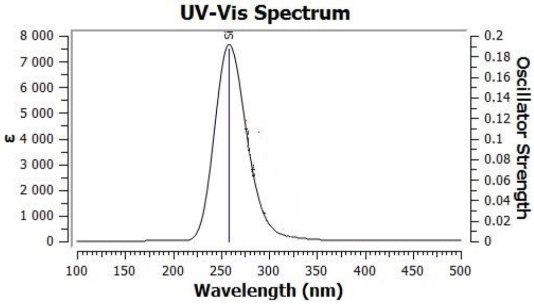

PtH_2ODACH-vitB6

Figure S1 (Continuation). Computed (MN15/def2-TZVP level of theory with PCM model and water as a solvent) UV-Vis spectra for selected Oxaliplatin complexes with nucleobases and B vitamins.

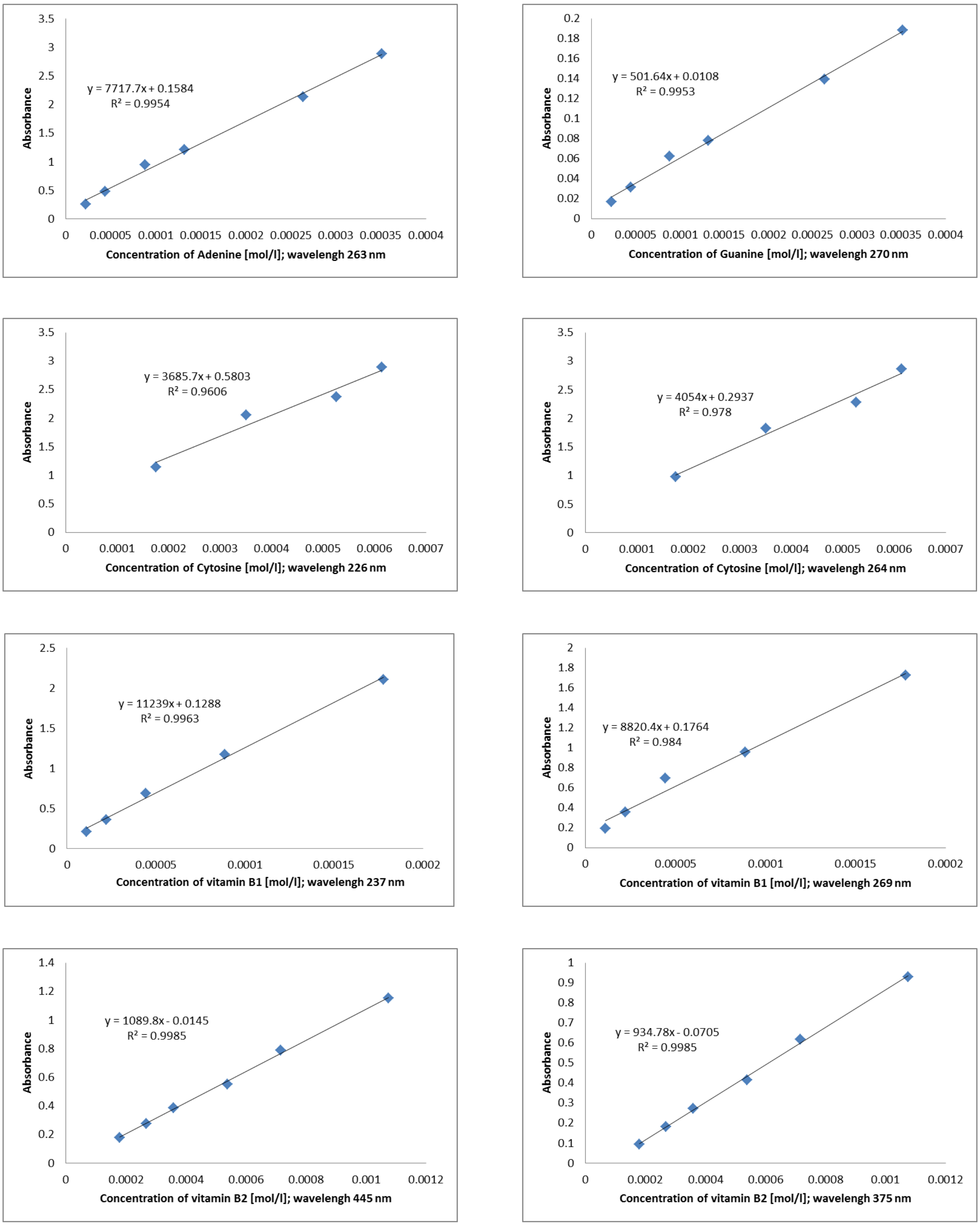

Figure S2. A standard curves for nucleobases and vitamins from B group.

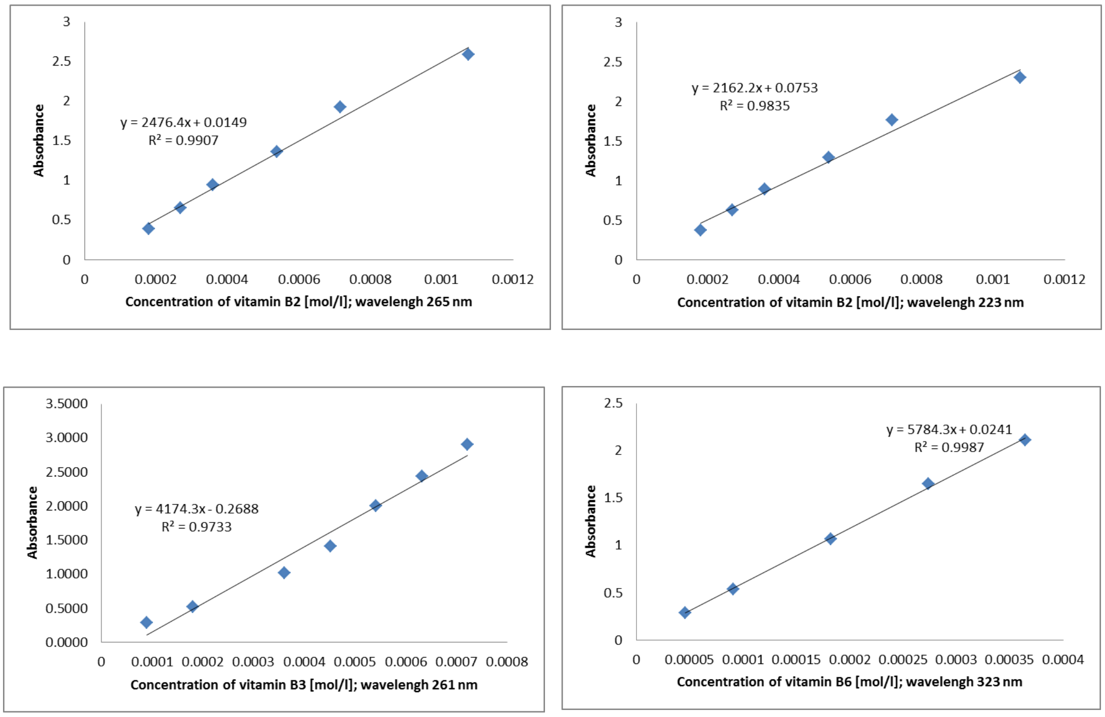

Figure S2 (Continuation). A standard curves for nucleobases and vitamins from B group.

Table S1. Computed (MN15/def2-TZVP level of theory with PCM) five excitation energies in eV and nm (for singlet-singlet transitions) and oscillator strength (in bracket) for ten selected complexes. The most intensive transitions are marked in red.

Singlet	Excitation energy [eV]	λ [nm]
	**PtClDACH-Ade**	
1	3.9897	310.76 (0.0008)
2	4.0580	305.53 (0.0002)
3	4.1143	301.35 (0.0007)
4	4.4468	278.81 (0.0003)
5	5.0904	**243.56 (0.2483)**
	**PtClDACH-Gua**	
1	3.9759	311.84 (0.0008)
2	4.0077	309.37 (0.0003)
3	4.0307	307.60 (0.0007)
4	4.3754	283.36 (0.0004)
5	4.8771	**254.22 (0.1861)**
	**PtClDACH-Cyt**	
1	3.9056	317.45 (0.0005)
2	3.9386	314.79 (0.0006)
3	4.0123	309.01 (0.0007)
4	4.3926	282.26 (0.0002)
5	4.9527	**250.34 (0.1697)**
	**PtH$_2$ODACH-Ade**	
1	4.0211	308.34 (0.0010)
2	4.2529	291.53 (0.0010)
3	4.2714	290.27 (0.0027)
4	4.4684	277.47 (0.0003)
5	5.1011	**243.06 (0.2614)**
	**PtH$_2$ODACH-Gua**	
1	4.0265	307.92 (0.0013)
2	4.1964	295.45 (0.0036)
3	4.2504	291.70 (0.0001)
4	4.5507	272.45 (0.0005)
5	4.7595	**260.50 (0.1784)**
	**PtH$_2$ODACH-Cyt**	
1	3.9082	317.24 (0.0014)
2	4.0883	303.26 (0.0018)
3	4.3372	285.86 (0.0006)
4	4.4406	279.21 (0.0005)
5	4.9486	**250.54 (0.1747)**
